# A bibliometric analysis of carbon neutrality: Research hotspots and future directions

**DOI:** 10.1016/j.heliyon.2023.e18763

**Published:** 2023-07-27

**Authors:** Xinru Xu, Xunjie Gou, Weike Zhang, Yunying Zhao, Zeshui Xu

**Affiliations:** a Business School, Sichuan University, 610064, Chengdu, China; b School of Public Administration, Sichuan University, Chengdu, 610064, China

**Keywords:** Carbon neutrality, Renewable energy, Carbon emission, Thematic evolution, Bibliometric

## Abstract

Global attention has shifted in recent years to climate change and global warming. The international community has set the objective of carbon neutrality to address the climate crisis. Carbon neutrality has drawn significant attention as a crucial step in the fight against climate change, with individual nations having established their carbon neutrality targets. This paper aims to use bibliometric analysis to investigate research hotspots and trends in carbon neutrality research, and accesses the literature through the Web of Science (WoS) core database and undertakes an in-depth examination of 909 publications linked to carbon neutrality around the world using Vosviewer and Bibliometrix software. According to the findings, the number of carbon neutrality publications has increased dramatically in recent years. There are also notable differences in carbon neutrality research across countries and regions. China and the US are the primary drivers and leaders of carbon neutrality research, and developing countries have relatively little carbon neutrality research. Research has concentrated on carbon neutrality’s practical, technical, policy, and economic aspects, as well as renewable energy sources, carbon conversion technologies, and carbon capture and storage technologies are also research hotspots. The paper also outlines opportunities for the advancement of carbon neutrality research in the future, including how it might be further integrated with Artificial intelligence (AI) and the metaverse, and how to attack the difficulties and uncertainties faced by the post-epidemic rebound. This study aids in understanding the current state of the field of carbon neutrality research and can be used to guide future studies.

## 1 Introduction

Human activities have recently caused significant changes in the atmosphere, land, oceans, and biosphere, and the rate of climate warming is increasing at a never-before-seen rate [1]. The greenhouse effect, acidification of water sources, and glacier melting are only a few of the environmental issues brought on by excessive CO_2_ emissions [2]. These issues have had a major impact on human social and economic life due to the increasing frequency and intensity of extreme weather. Therefore, a prevalent concern for academics at this point is how to attain minimal carbon emissions in all facets of industry, life, and socioeconomic development.

At the Paris Climate Change Conference (PCCC), which took place on December 12, 2015, 197 UNFCCC (The United Nations Framework Convention on Climate Change) members came to an agreement to adopt the Paris Agreement [3], which lays out a global action strategy to combat climate change after 2020. This agreement was made in response to increasing global greenhouse gas emissions and global temperatures. To lessen the risks and effects of climate change, the agreement sets a goal of maintaining the global average temperature rise compared to pre-industrial levels to within 2 °C by the end of the century and for reducing the rise to under 1.5 °C [4]. A new system of climate governance is established by the Paris Agreement through a nationally owned contribution method, and nations are gradually implementing their own “carbon peaking” and “carbon neutrality” targets. Carbon peaking is the term used to describe a point in time when CO2 emissions cease increasing, reach their peak, and then begin to decline. And carbon neutrality refers to the ability to achieve a positive or negative offset through the planting of trees, energy conservation, and emission reduction to achieve a relative “zero emissions” for the total amount of carbon dioxide or greenhouse gas emissions produced directly or indirectly by a nation, enterprise, product, activity, or individual within a specific time frame [5]. As a result, in the current context, the urgent need for environmental protection has led to a broad interest in and quick development of the topic of carbon neutrality, with a corresponding explosion in the body of literature. Leading academics from other domains are increasingly exploring and researching the multidisciplinary field of carbon neutrality, which involves knowledge from many different disciplines and complicated problems.

It is important and fruitful to methodically analyze and consider the findings that have already been achieved in this subject as theoretical research and practical experience have accumulated [6]. Some overviews of carbon neutrality from specialized perspectives have previously been presented by researchers from related fields in several research areas, including studies on the use of environmentally friendly materials [7], difficulties in high carbon emission industries [8,9], carbon-neutral technologies [10,11], and energy transition pathways [12], etc. The field of carbon neutrality is advanced by these publications, which offer a thorough summary of the disciplinary knowledge within it. The purpose of this paper is to review the carbon neutrality literature published to date, conduct a bibliometric analysis as well as a content analysis using visualization tools, and answer the following three research questions:

(1) What are the characteristics of the publication trend, the keyword developments, the authors, the nations/regions, institutions, as well as the collaborations between them, for papers on carbon neutrality?
(2) What are the main hot spots in the field of carbon neutral research and what are the ways to achieve it?
(3) What are the foreseeable directions for research?

For these reasons, by Vosviewer and Bibliometrix software, the collected literature data with carbon-neutral titles are visualized and analyzed to give a full picture of the field’s dynamic evolution and trends in terms of descriptive statistical analysis (high-contributing authors, journals, institutions and countries), collaborative network analysis, and keyword clustering analysis, respectively. This is followed by a discussion of potential future study directions in the area, and lastly the work completed in this paper is summarized and the future advancements of carbon neutrality research is prospected.

The remainder of the paper is structured as follows: Section 2 describes the bibliometric visual analysis preparation in terms of data acquisition and tool selection. Section 3 shows bibliometric analysis results including the publication trends, and the analyses by authors, countries/regions, institutions, categories, and keywords. Section 4 goes into detailed discussion about the key themes gained from the bibliometric of the literature and suggests further research directions of investigation. In Section 5, we provide a summary of the research presented in this paper.

## 2 Methodological approach

The bibliometric can assist scholars in quickly grasping the development pulse as well as the border hotspots of the relevant academic fields through a quantitative, systematic assessment of the published literature. To achieve the suggested objective, this research conducts a complete bibliometric analysis of the literature in the field of carbon neutrality.

### 2.1 Data acquisition and processing

In this study, we chose the Web of Science (WoS) as the database source for the studied literature after evaluating each database’s authority, informational richness, and compatibility with different bibliometric analysis software. For the bibliometric review, publications having the phrase “carbon neutrality” or “carbon-neutral” in their titles are chosen as the most pertinent papers, the search rules are presented below. We initially look through the WoS core collection database for publications with the phrase “carbon neutral” or “carbon neutrality” in their titles, limiting the publication period to between 2000 and 2022 (ending December 16, 2022). Then, only “theses”, “review papers” and “online publications” are allowed. During the collection procedure, the irrelevant material is simultaneously evaluated, and a total of 909 publications are finally screened out. For the final bibliometric analysis, the abstracts, years, keywords, authors, institutions, countries and references of these articles are exported to text files.

### 2.2 Research methods

Visual analysis techniques of literature are frequently employed in scientific research since they enable us to swiftly extract useful information from the literature that is pertinent to a given field of study. Numerous feature-rich literature analysis software is quickly appearing due to the rapid development of their application, including Citespace, VOSviewer, HistCite, Bibliometrix, etc. In this study, Bibliometrix and VOSviewer will serve as the primary tools for visualizing each topic’s fundamental metrics and the network linkages that connect them from various angles. We examine the relationships between nodes, which stand in for authors, organizations, partnerships, keywords, etc., by the visual analysis feature of VOSviewer, to discover future research hotspots and trends. Furthermore, we make use of the Bibliometrix tool to offer VOSviewer capabilities like topic maps and factor analysis that VOSviewer is missing.

## 3 Results

In this subsection, we quantitatively analyze the literature with the assistance of tools like VOSviewer and the Bibliometrix R-package to map the knowledge landscape of carbon neutrality. Then, we display the trends in the volume of literature, the distribution and development of important subjects, the citation status, and the excellence of various nations/regions, organizations, journals, and authors in the field of carbon neutrality since 2000 with the use of numerous graphs.

### 3.1 Publications and citations trend

**Fig. 1:**
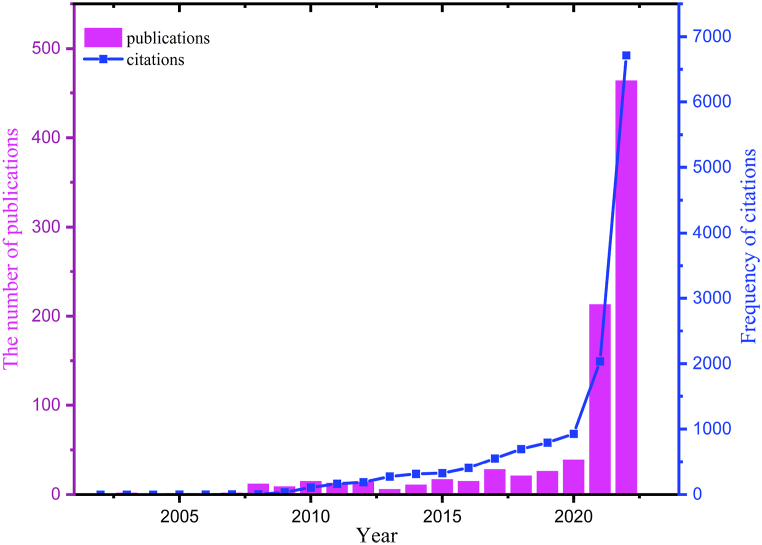
Number of publications and citations from 2000 to 2022 (by December 16, 2022).

Fig. 1 depicts the development in the number and citations of publications examining carbon neutrality. As can be seen, until 2020, the amount of literature and citations in this discipline indicate a comparatively constant trajectory. Since 2020, when the number of publications climbed from 38 to 448 in 2022, the number of publications on carbon neutrality has demonstrated an enormous growth trend. The first citation appeared in 2005, however, publications were cited 6455 times in 2022 alone (By December 16, 2022), indicating that the field of carbon neutrality is developing at a previously unheard-of rate. Due to the increased importance and seriousness of environmental preservation, as well as China’s announcement in September 2020 that the nation aims to achieve “carbon neutrality” by 2060, the field of carbon neutrality has attracted more attention in recent years and articles published in the 2022 have accounted for more than half of the total number of articles. As a result, we may anticipate that this field will continue to yield fruitful outcomes in the future, contributing to the world’s efforts to attain carbon neutrality.

Next, we examine the impact of publications in the field by performing further analysis of highly cited publications. First, as shown in Table 1, the top 10 cited publications on the topics of carbon neutrality are listed. Publications rated 1, 2, 3, 6, 8, 9, and 10 are in the discipline of chemistry, publications ranked 4,5 are in the field of environmental science, and publications ranked 7 are in the field of agricultural bioenergy. In 2009, the top-ranked publication proposed an inventive strategy for carbon neutrality conversion from harmful greenhouse gases to carbon-neutral fuels and synthetic hydrocarbons, and this strategy has received numerous citations, and it also involves converting greenhouse gases into beneficial carbon fuels [13]. The second-ranked piece of literature examines methods for switching from a fossil fuel economy to a hydrogen economy and emphasizes the importance of carbon-neutral technologies and fuels in the process [14]. Similar to the second-ranked publication, the third-ranked one focuses on metal-organic frameworks to lessen reliance on fossil fuels and create methods and technologies for the capture, storage, transport, and conversion of carbon dioxide gas molecules [15]. Notably, the paper with the highest average number of citations per year, which is ranked fifth, garnered 192 citations after being published online in October 2021. Based on worldwide data, the publication contrasts the disparity in carbon neutrality between China, the EU, and the US, evaluates China’s difficulties in becoming carbon neutral, and offers specific strategic advice. The outcomes they produced have been acknowledged by academics and have gotten a lot of citations. In addition, other publications in Table 1 provide light on the development of sustainable energy technologies [16], the synthesis of carbon-neutral fuels [17], as well as the difficulties, solutions, and future of the transition to carbon neutrality [18,19]. They have made major contributions to the growth of the carbon neutrality field with their study on subjects like renewable energy, carbon neutral fuels, carbon capture, and cutting-edge technologies.

**Table 1:** Top 10 most cited publications.

Rank	Title	Year	Source	Citations Numbers	Average Per Year
1	Chemical recycling off carbon dioxide to methanol and dimethyl ether: from greenhouse gas to renewable, environmentally carbon neutral fuels and synthetic hydrocarbons	2009	Journal of Organic Chemistry	1024	73.14
2	“Green” path from fossil-based to hydrogen economy: an overview of carbon-neutral technologies	2008	International Journal of Hydrogen Energy	598	39.87
3	The role of metal-organic frameworks in a carbon-neutral energy cycle	2016	Nature Energy	286	40.86
4	Goodbye to carbon neutral: Getting biomass footprints right	2009	Environmental Impact Assessment Review	194	13.86
5	Challenges toward carbon neutrality in China: Strategies and countermeasures	2021	Resources Conservation and Recycling	192	96
6	Carbon-neutral sustainable energy technology: Direct ethanol fuel cells	2015	Renewable & Sustainable Energy Reviews	181	22.63
7	Is woody bioenergy carbon neutral? A comparative assessment of emissions from consumption of woody bioenergy and fossil fuel	2012	Global Change Biology Bioenergy	174	15.82
8	Integrative CO2 Capture and Hydrogenation to Methanol with Reusable Catalyst and Amine: Toward a Carbon Neutral Methanol Economy	2018	Journal of the American Chemical Society	154	30.8
9	Carbon dioxide Fischer-Tropsch synthesis: A new path to carbon-neutral fuels	2017	Applied Catalysis B-Environmental	143	23.83
10	Technologies and perspectives for achieving carbon neutrality	2021	Innovation	136	68

### 3.2 Characterization of the authors of the publications

The authors, regions/countries, and institutions of publications are systematically analyzed in this subsection to find the most productive and significant authors, regions/countries, and institutions. This paper uses the number of publications as the primary index to measure the contribution of authors. The top 5 authors are shown in Table 2 along with their total number of publications, the number of publications (NP), the total number of citations (TC), and influence h-index. As we can see, Umar, Muhammad is at the top of the list with 7 publications, 435 citations, and the highest impact 7, and holds a key position in the field. Notably, with 5 publications and a whopping 1290 citations, Prakash, Surya has delved into chemistry and materials science, as well as themes like carbon dioxide capture and green fuels, applying expertise from various disciplines to the field of carbon neutrality and encouraging the construction of a carbon neutral economy around the world.

**Table 2:** Top 5 most publications research scholars.

Rank	Name	NP	TC	h-index
1	Umar, Muhammad	7	435	7
2	Adebayo, Tomiwa Sunday	7	126	5
3	Kirikkaleli, Dervis	6	272	6
4	Cui, Qiang	5	91	4
5	Prakash, Surya	5	1290	5

**Fig. 2:**
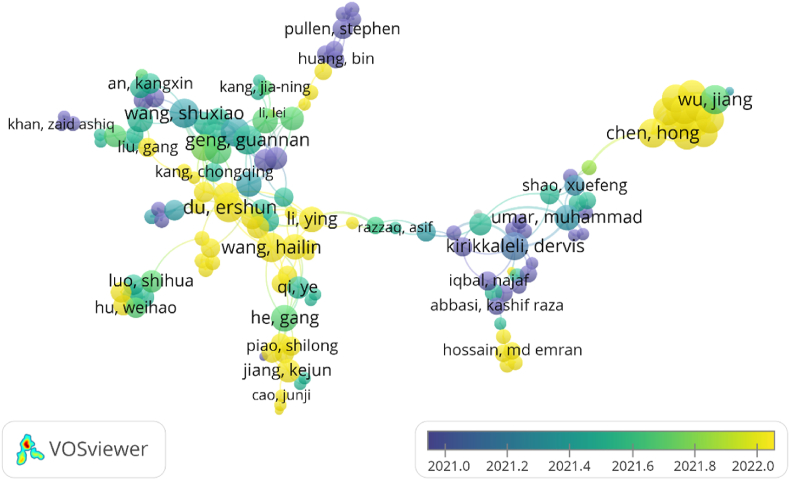
Authors’ cooperation network.

Collaboration among authors in a field aids in the production of more exceptional works, and we then utilize VOSviewer software to visually examine collaboration among authors in the area. Fig. 2 depicts the graph of author collaboration, where the larger corresponding nodes in the collaboration network indicate a greater number of co-authored publications with other authors. The 143 authors with at least two publications are chosen for analysis and grouped into 13 clusters, where Cluster 1 including 19 authors and Cluster 13 containing just 3 authors in descending order. Fig. 2 depicts the graph of an author collaboration network with a temporal dimension. Overall, the cooperation network in this sector has progressively formed and swiftly spread outward since 2021, showing more frequent collaboration among authors as the number of publications has increased dramatically. The authors with the most publications, Umar, Muhammad and Adebayo, Tomiwa Sunday have excellent collaborative network nodes and are actively looking for collaborations to expand their research. At the same time, new writers are appearing, and we anticipate that more academics will join the area to further its advancement in the future.

### 3.3 Characterization of the sources of the publications

**Fig. 3:**
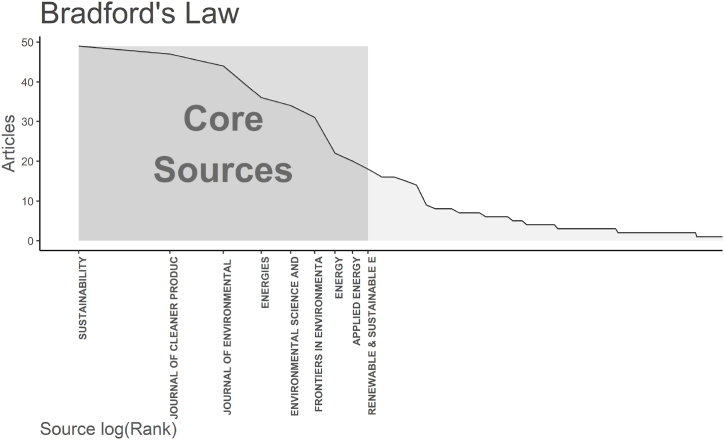
Source clustering through Bradford’s Law.

With the aid of bibliometric software and visual analysis techniques, this subsection offers a systematic examination of the publication’s source journals to identify the most significant and representative source journals. Fig. 3 displays the outcomes of Bradford’s law source clustering, which aims to graphically represent the distribution of documents in a field among several journals and from which we can understand which journals the area's pertinent literature is concentrated. The 909 publications retrieved in this paper are from 353 journals, the core area of the source journals in this discipline, as shown in Fig. 3, has 9 journal sources, namely “*Sustainability*” (49), “*Journal of Cleaner Production*” (47), “*Journal of Environmental Management*” (44), “*Energies*” (36), “*Environmental Science and Pollution Research*” (34), “*Frontiers in Environmental Science*” (31), “*Energy*” (22), “*Applied Energy*” (20), and “*Renewable & Sustainable Energy Reviews*” (18).

The value and effect of a journal, however, are also directly correlated with measures like citations and are not just determined by the number of publications. This subsection then examines the total number of citations made by the journals to discover the most significant sources. The top 10 journals are listed in Table 3 along with their TC, NP, impact factor (IF), and debut publication year. The majority of the journals listed in Table 3 are in the environmental sciences and energy fuels fields. We can see that the “*Journal of Environmental Management*” received 1653 citations for 44 publications, demonstrating that the journal pays attention to both the quantity and quality of publications in the field. It is important to note that several journals, such as *International Journal of Hydrogen Energy* and *Journal of Organic Chemistry*, have only a few articles but are nevertheless highly regarded in the field of carbon neutrality and earn a significant amount of citations. IF_2021_ represents the absolute strength of the journal in the field it covers, and there is no shortage of high-IF journals in the field, such as *Nature Energy*, which boasts an IF of 67.44.

**Table 3:** Top 10 most contributive journals based on TC.

Rank	Source	TC	NP	Initial year	IF_2021_
1	Journal of Environmental Management	1653	44	2012	8.91
2	Journal of Organic Chemistry	1021	1	2009	4.19
3	International Journal of Hydrogen Energy	615	3	2008	7.19
4	Journal of Cleaner Production	565	47	2009	11.07
5	Renewable & Sustainable Energy Reviews	465	18	2015	16.79
6	Applied Energy	457	20	2016	11.45
7	Resources Conservation and Recycling	377	14	2021	13.71
8	Energy Policy	314	8	2007	7.58
9	Energy	298	22	2012	8.86
10	Nature Energy	287	2	2016	67.44

Next, we concentrate on the research institutions of publications. Understanding the research institutions that are now making significant contributions and improving institutional cooperation are aided by analyzing the research institutions of publications. Table 4 lists the top 5 research institutions according to the number of publications along with their output, the year of initial publication, nation, percentile of 909 articles, and h-index. It is worth noting that the top five publishing institutions are all from China. Chinese Academy of Sciences, with 52 publications, is the institution with the most publications, followed by Tsinghua University, with 46 articles, all of which have received numerous citations. They have been involved in studying various aspects of carbon neutrality, including developing technologies for carbon capture and storage, promoting renewable energy sources, and conducting assessments of greenhouse gas emissions, and have had a significant impact on the field. They have been involved in studying various aspects of carbon neutrality, including developing technologies for carbon capture and storage, promoting renewable energy sources and conducting assessments of greenhouse gas emissions, and have had a significant impact on the field.

**Table 4:** Top 5 institutions with the most publications.

Rank	Institutions	NP	NC	Country	Percentile of 909	Initial Year	h-index
1	Chinese Academy of Sciences	56	657	China	6.161	2015	13
2	Tsinghua University	46	676	China	5.061	2021	15
3	North China Electric Power University	23	112	China	2.53	2016	6
4	University Of Chinese Academy of Sciences Cas	22	357	China	2.42	2016	7
5	China University of Mining Technology	18	155	China	1.98	2021	6

**Fig. 4:**
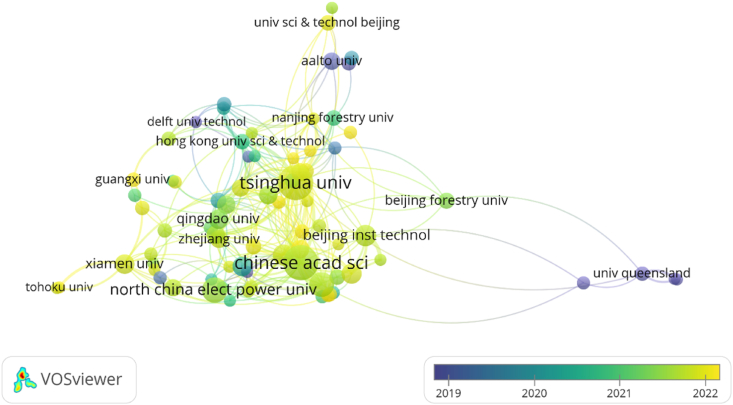
Institutions cooperation network.

The next step is to choose the research institutes that has more than five publications for collaborative network analysis. In Fig. 4, the node sizes indicate how frequently they work with other universities, and a cluster is represented by a node of the same color. As we have seen, Tsinghua University and the University of Chinese Academy of Sciences have a large number of publications, have developed strong relationships with other institutions and are at the core of collaborative networks that are steadily growing their influence in the field. Tsinghua University collaborates with the University of Chinese Academy of Sciences and other research institutions and organizations both within China and internationally to share knowledge and expertise in the field of carbon neutrality. These collaborations aim to advance research, develop innovative solutions and contribute to global efforts in mitigating climate change. We can observe that there is close cooperation between institutions in the China region of the network, and the institutions in other regions of the cooperative network are marginally inferior in comparison to the number. Scholars from various institutions should actively interact and exchange ideas to promote the rapid achievement of global carbon neutrality since cross-regional institutional exchanges and collaborations have a very favorable impact on encouraging research in this area.

### 3.4 Characterization of the regions/countries of the publications

**Fig. 5:**
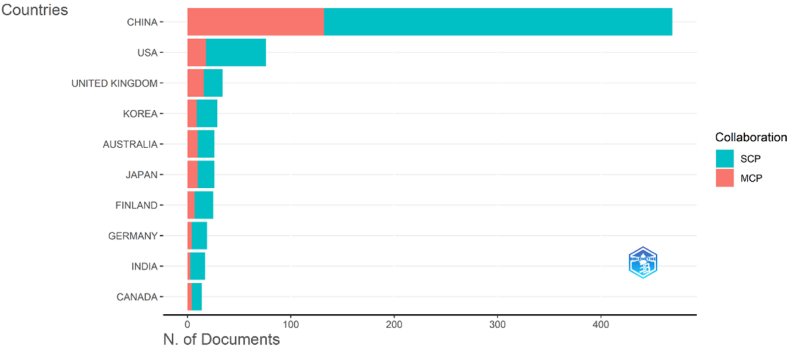
Top 10 most prolific countries/regions (SCP: Single Country Publications, MCP: Multiple Country Publications).

The fundamental method for comprehending the global distribution and development of research in the field of carbon neutrality is to examine national/regional collaboration, publishing indicators, etc., in the area. The top 10 nations in terms of publications are shown in Fig. 5, where MCP denotes the number of papers co-authored by authors of different nations, and SCP denotes the number of papers co-authored by authors of the same nation. Overall, the authors prefer working with other scholars from their nation to working with those from other countries. China is considerably ahead of other nations in terms of publications, placing first with 469 articles (SCP: 337, MCP: 132). In China, the first publication debuted in 2012, and the years that followed demonstrated a consistent growth pattern. However, a significant increase in publications occurred in 2021 and 2022, with 136 and 352 publications, respectively. This phenomenon’s emergence is closely correlated with Chinese national policy. Chinese academics are now more enthusiastic about carbon neutrality and aware of technological innovation as a result of the introduction of China’s “carbon peaking and carbon neutrality” target. In addition, we can also see that, of the top 10 nations, 8 are developed nations and 2 are developing nations. The urgency of reducing emissions differs from nation to nation, emission reduction is a pressing challenge for developed nations, and they have decades of experience in the field of carbon reduction, but the majority of developing nations still prioritize economic growth. To avoid detours on the path to emission reduction, developing nations should learn more about and draw lessons from the governance experience of developed countries with leading carbon neutrality ambitions, and continue optimizing policies and actions.

Whether international cooperation between diverse nations/regions has a direct impact on the rapid future development of this field. We undertake a visual study of the collaboration and exchange between nations/regions to investigate this issue. North American and Asian nations are interconnected strongly. China and the US have established a global network of collaboration, with China also advancing tighter ties with the US (frequency: 42), the UK (26), Australia (21), Pakistan (19) and Turkey (16). Additionally, the US has established tight ties with nations including China, the UK (9) and Australia (7). The exchanges between nations in Africa and South America and nations in other regions, however, are rather meager, and the collaboration is also infrequently fostered. Scholars from all nations should actively cooperate and exchange ideas to actively promote the development of global carbon neutrality, protect the environment of the entire globe, and minimize carbon emissions. To reduce their environmental effect while expanding their economies, developing nations must actively learn from the experiences of developed nations and carry out more worthwhile, important, and high-quality research.

### 3.5 The thematic distribution and evolution of publications

This subsection firstly presents a cursory to an in-depth review of publications’ themes in the field of carbon neutrality, delving into research hotspots and trends in this area. Referring to the results of the subject analysis provided in the WoS search result analysis, Table 5 shows the number of publications and the percentile of the top 10 hot research directions. As we’ve seen, publications in the field of carbon neutrality are often interdisciplinary, bringing together knowledge from several disciplines such as environmental engineering, architecture, chemistry, civil engineering, etc. Publications in the fields of environmental science and energy fuels make up the highest share among them, totally accounting for up to 63.69%, and are closely followed by research in green sustainability, science and technology. This demonstrates that the research on carbon neutrality is closely related to the environment, energy and technology, so how to manage energy carbon emissions, enhance energy fuels, and achieve sustainable development are crucial areas for study.

**Table 5:** Top 10 research directions.

Rank	Research directions	Number of records	Percentile
1	Environmental Sciences	352	38.72%
2	Energy Fuels	227	24.97%
3	Green Sustainable Science Technology	178	19.582%
4	Environmental Studies	115	12.65%
5	Engineering Environmental	92	10.12%
6	Engineering Chemical	51	5.61%
7	Construction Building Technology	35	3.850%
8	Thermodynamics	35	3.850%
9	Engineering Civil	34	3.74%
10	Chemistry Multidisciplinary	31	3.41%

**Fig. 6:**
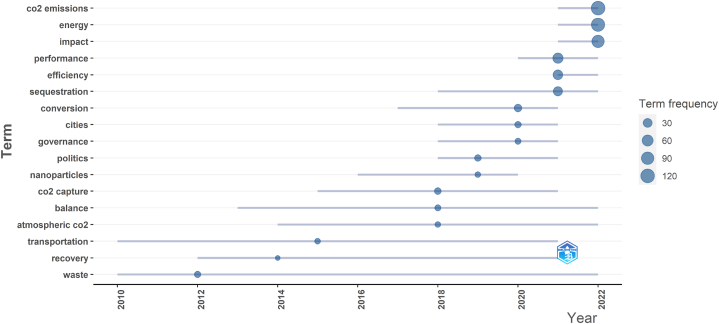
Trend of topics.

The findings of aforementioned analysis provide a thorough overview of the major areas for future research in the field of carbon neutralization, and they also provide us with a basic direction of the range of topics that have been discussed. We can quickly comprehend the development of research in the area of carbon neutrality and thoroughly grasp the hot spots in different periods by looking at the relationship and development status between highly frequent co-occurring keywords, which serves as a guide for our future research. The development pattern of the sub-themes in this field is then examined using the bibliometric and VOSviewer tools. The findings of our subsequent application of the Bibliometrix tool’s TrendTopics module to further examine the development trend of keywords are displayed in Fig. 6. The size of blue nodes in Fig. 6 denotes the frequency of related keyword, and the length of line segment represents the year covered by the high frequency of keyword. We can see that the trend of high-frequency keywords has gradually changed since 2010, the high-frequency keywords are mainly concentrated in the years 2020–2022, which suggests that these two years are the field’s expansion and deepening phase of development. Fig. 6 indicates that the early publications of the study concentrated more on waste management and recycling [20], GHG emissions from the transportation sector [21], to support carbon neutrality. In the middle of development, the scope of carbon neutrality research was gradually narrowed to cities, and city carbon neutrality was born [22]. According to the study conducted thus far, the reduction of CO_2_ emissions, the search for renewable alternative energy sources [23], and energy usage performance and efficiency [24], have all emerged as hot research areas.

**Fig. 7:**
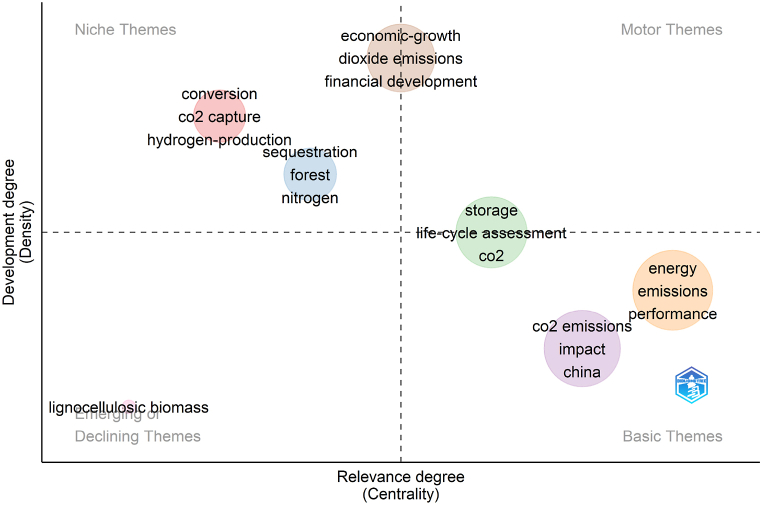
Thematic map of keywords.

The distribution of keywords following clustering is seen in Fig. 7. The horizontal axis indicates the importance level and the vertical axis indicates the development level. Fig. 7 is divided into four quadrants, with the first quadrant representing niche topics with low importance but high development level, the second representing popular topics with high importance and high development level, and the third and fourth representing declining topics and basic topics, respectively. The keywords in this domain are separated into six clusters, and the figure shows only the top three ranked keywords in terms of frequency in the clusters. The six categories of keywords are mainly concentrated on niche topics and basic themes. CO_2_ emissions, impact studies, China’s “carbon peaking and carbon neutrality” target, and renewable energy are some of the more important research topics, but they are still in their infancy, with plenty of room for innovation. Among them, the research of niche themes in the first quadrant, such as “CO_2_ capture”, and “hydrogen production”, are now at an advanced stage, allowing for future attention on other topics, especially the basic themes. It can be seen that research on technologies related to carbon capture and storage, carbon conversion, and the production of hydrogen energy has attracted considerable attention and achieved significant success. These technologies, along with other carbon-negative technologies, are crucial tools for achieving the goal of carbon neutrality.

## 4 Finding and discussion

Due to the changing climate and the requirements of global policy, the literature on carbon neutrality has recently seen an exponential development phase. This has drawn businesses and academics from all over the world to the subject. Through the literature bibliometric analyses, we can learn a lot from it.

### 4.1 Lesson learned in bibliometric analysis

1. This paper provides a bibliometric analysis of 909 publications in the WoS core database with the title contains “carbon neutrality.” The first publication was published in 2005, and since 2020, there has been a sharp increase in the number of publications, with publications from 2022 alone accounting for more than half of the 909 publications (By December 16, 2022).
2. With 7 publications and 435 citations, Umar, Muhammad come in first place on the list of top contributing authors, followed by Adebayo, Tomiwa Sunday. Based on the relationships among authors who had at least two publications, 143 authors are divided into 13 clusters, in which Umar, Muhammad and Adebayo, Tomiwa Sunday hold the key places in the collaborative network.
3. The selected 909 publications come from 353 journals, with *Sustainability*, *Journal of Cleaner Production* and *Journal of Environmental Management* being the top three journals in terms of the number of publications. The top 5 publishing research institutions are all located in China, and there is a strong network of collaboration between institutions, with Tsinghua University and the Chinese Academy of Sciences serving as the key nodes.
4. China is the most prolific nation, with 469 publications, followed by the US and the UK. Among the top 10 nations in terms of publications, there are considerably more developed countries than developing countries. The development of this field has been substantially aided by the strong cooperation that exists between China and the US as well as the close cooperation that has been created with other nations.
5. The study of carbon neutrality is an interdisciplinary field that draws on knowledge from several fields, including chemistry, architecture, environment, etc. The results of the word cloud analysis demonstrate that energy use and carbon emissions are the primary themes in this area and that these themes are evolving and developing as new technologies for carbon capture, carbon conversion, carbon reduction, and green hydrogen are developed.

### 4.2 Discussion

The protection of the environment and hastening the achievement of carbon neutrality are currently the shared goals of all humankind, but the process faces numerous hurdles, including energy structure, resource constraints, and technological limitations. The research hotspots and main routes to carbon neutrality are carbon replacement, carbon reduction, and Carbon Capture, Utilization and Storage (CCUS), according to the bibliometric analysis. This subsection gives the following discussions and indicates some expectations for future research based on what has been discussed.

#### 4.2.1 Carbon replacement and carbon neutrality

Carbon replacement refers to the substitution of renewable energy sources for fossil fuels. Energy is a resource that is essential to all industries, and the tremendous amount that is consumed puts an enormous strain on the environment and governments [25]. Therefore, there will need to be an energy transition and a search for new, clean, and renewable energy sources if we want to reach carbon neutrality in the allotted period. Clean energy sources such as wind, solar, hydro, biomass and geothermal energy are included in the category of renewable energy. In addition, due to their low resource consumption and low pollution risk, nuclear and hydrogen energy have been designated as crucial essential avenues for reaching “carbon neutrality” and ensuring national energy security. Several studies have examined the connection between renewable energy and carbon neutrality. Renewable energy can help achieve carbon neutrality by reducing CO_2_ emissions. Das et al. [26] used the quantile ARDL model to analyze the use of renewable energy in India and found that for every 2% increase in renewable energy use, there is a 1% decrease in CO_2_ emissions. Using the method of moments quantile regression (MMQR), Liu et al. [27] evaluated the contribution of renewable energy consumption to carbon neutrality in the BRICS countries and found that the use of renewable energy reduced CO_2_ emissions in all quartiles in the BRICS countries. In connection to the US carbon neutrality aim, Zhao et al. [28] discovered that environmental and renewable energy R&D positively contribute to carbon neutrality by reducing atmospheric CO_2_ emissions, while renewable energy R&D also plays a significant role in lowering haze pollution like PM2.2. As a result, one of the most important ways to guarantee that carbon neutrality goals are realized is to increase expenditure and investment in research and development for renewable energy sources.

However, certain renewable energy sources are highly unpredictable and non-adjustable, resulting in low utilization of renewable energy and having an adverse effect on the power system’s ability to operate safely [29]. To promote the high rate of renewable energy use, the power system must be fully optimized, and each nation should establish a renewable energy-based power system that fully supports the development and consumption of renewable energy in terms of energy storage, smart grids, demand side, and dispatch [30]. Guo et al. [31] integrated 5G base stations into the microgrid to participate in demand and jointly dispatched the distributed clean energy generation, micro gas turbines, and energy storage systems in the microgrid to help increase the effectiveness of renewable energy use. Takahashi et al. [32] created intelligent power modules that combine versatility and intelligence to instantaneously react to varying power sources like solar and wind power and respond to the fast-growing renewable energy industry. However, more research is needed to accelerate the replacement of fossil fuels with renewable energy, and the trends in renewable energy production, scalability of energy storage, and cost-effectiveness still remain significant challenges. Additionally, many problems still need scientific, social, political, and technological solutions in order to move the global community toward carbon neutrality.

#### 4.2.2 Carbon emission reduction and carbon neutrality

Excessive carbon emissions are a major contributor to global warming and are harming the environment in which people live permanently. What exactly are the factors that contribute to reducing CO2 emissions? Liu et al. [33] demonstrates through empirical investigations that while GDP has a major positive effect on CO2 emissions, the establishment of an international trade tax, low energy intensity, and numerous internet users all contribute to reducing CO2 emissions. Meanwhile, when analyzing the variables affecting carbon emissions, the Logarithmic Mean Divisia Index (LMDI) model is frequently used, taking into account factors like industrial structure, energy intensity, energy efficiency, and economic growth [34]. Research on the factors that influence carbon emissions for various regions is essential for regional carbon reduction and achieving regional carbon neutrality.

Individual nations, regions, and cities must put comprehensive, multi-level, and multi-scale emission reduction strategies into place for all sectors to become carbon neutral. Yao et al. [35] performed a bibliometric analysis of the frontier directions of carbon emission reduction, in which some policies and practices used by various nations and sectors to reduce carbon emissions were analyzed, and several pathways and development models for emission reduction were examined. The construction industry is the largest user of resources and energy in the world, and its ability to cut emissions will have a huge impact on the transition to carbon neutrality [36]. The impact of decarbonization decisions in the planning, delivery, closure, and operational phases of construction projects was evaluated using a qualitative approach that combines a systematic literature review with in-depth semi-structured interviews with experts to encourage carbon reduction in the construction industry [37]. Researchers are interested in the steel industry since it is challenging to cut emissions. Blast furnaces powered by coal are used to make steel, which produces a lot of carbon emissions, Yu and Tan [38] thought that the steel industry had to apply ground-breaking technologies like carbon capture and storage and hydrogen-based steelmaking more quickly. Li and Hanaoka [39] indicated that China, the world’s largest steel producer, might change steel output and the energy mix through the use of a combination of tax policies, environmental rules, and low-carbon power generation. The power sector also has a crucial role to play in achieving carbon neutrality goals, and the interaction and integration of the power and carbon markets will help to more effectively reduce carbon emissions, with their institutional relevance, price interactions, and trading behavior having an impact on decision-making and carbon reduction [40]. Therefore, it has been a hot research topic to continuously study the factors influencing carbon emissions and find the pathways to reduce emissions in high carbon emitting industries, in order to propose useful and efficient solutions to achieve carbon neutrality.

#### 4.2.3 CCUS and carbon neutrality

CCUS is an important technology for reducing carbon emissions and can be significant in the process of achieving carbon neutrality. It can offer essential solutions to decarbonize sectors like steel, chemicals, cement, and non-ferrous metals where it is challenging to reduce carbon emissions [41]. Cutting-edge CCUS technologies include Direct air capture (DAC), Flexible metal-organic frameworks (MOFs), Integration of CO_2_ capture and conversion (ICCC) and Electro-catalytic CO2 reduction (ECR), these cutting-edge CCUS technologies are growing in significance and influence [42]. According to Wei et al. [43], the contribution of CCUS to China’s crude steel industry will be in the range of 17.6%–56.5%, and the total cost will be between 189 billion USD and 633 billion USD, considerably aiding the green transformation and development of China’s steel industry. According to research by Chen et al. [44], the implementation of CCS technologies in emission-intensive businesses might avert 32% of Mexico’s GHG emissions in 2050, assisting Mexico in meeting its goal of reducing its overall GHG emissions.

Even though the technology is attracting a lot of interests from academics throughout the world as a carbon-negative technology, the risks and uncertainties related to deployment planning in support of achieving carbon neutrality have not yet been adequately addressed. Chen et al. [45] investigated the potential challenges that might result in a mismatch between the rate of CCUS deployment and the requirement to meet carbon neutrality targets. Findings show that high project failure rates, a lack of financial support, market incentives, insufficient regulatory frameworks and risk-sharing mechanisms, as well as insufficient exploration of geological storage capacity, are major obstacles to the deployment and optimization of CCUS. Additionally, the absence of CCUS-related legislation and regulations as well as a lack of adequate financial and human resources might represent serious obstacles to the growth of the CCUS sector [46]. Consequently, the directions that should be pursued to remove the obstacles in the planning and deployment of CCUS technology and to facilitate its adoption and practice globally are the development of technical standards and regulations, the creation of an enabling technical environment for enterprises, the improvement of the regulatory framework, and the acceleration of technology development [47].

### 4.3 Future research directions

With the growth of literature, the concept of carbon neutrality, which embodies the goal of preserving the environment and achieving sustainable development, has expanded and developed. Carbon neutrality encompasses an intricate body of knowledge from multiple disciplines and there are so many questions to be explored and resolved. By presenting the discussion of future visions below, this subsection expands on the previous discussions and outlines expectations for forthcoming research.

#### 4.3.1 Uncertainty in the exploration of transformation routes

One of the commonly used methods for projecting carbon emissions is the use of integrated assessment models, which concentrate on modeling key dynamics of the links among the energy, economic and climatic systems, and researchers simulate carbon emissions under various transition pathways using a variety of scenario analyses in combination with various models. The top-down model and the bottom-up model are two different modeling concepts and modeling frameworks that are incorporated into the integrated assessment model. Top-down models include CGE, RICE and MERGE models, while bottom-up models include MESSAGE, AIM and LEAP models, etc. [48]. The model takes into account several variables, such as technology, GDP, population, urbanization, energy efficiency, etc., that could have an impact on the carbon neutrality process. The process of achieving carbon neutrality under various scenarios can be examined by changing the values of various parameters. Yet, due to differences in model construction and data sources, there is a significant level of uncertainty in the models, and the outcomes vary. However, it can be challenging to quantify and imprecise to explain in terms of parameters when evaluating how policies affect carbon neutrality. More research is continually required to minimize the influence of these uncertainties, which have an impact on the results of model simulation [49].

Additionally, in today’s world, the process of recovering from the epidemic’s effects on the economy is unstable, there is uncertainty surrounding the relations between major powers, and the full impact of climate change has not yet been felt. All of these uncertain factors will have an impact on the transition to carbon neutrality. As a result, it’s critical to be proactive in addressing uncertainty in the process of reaching carbon neutrality, to raise awareness of the situation's ambiguity, and to consider a variety of uncertainties in the study.

#### 4.3.2 AI for carbon neutrality

In recent years, the use of AI is changing every aspect of humanity and is becoming increasingly important in driving the process of carbon neutrality. In the face of the complexity and uncertainty in carbon neutrality energy systems, traditional techniques have struggled to appropriately handle the ambiguity and uncertainty involved. Key developments in carbon-neutral green technology, such as hydrogen generation technologies [50], forecasting technology [51], and diagnostic monitoring technology [52], can be facilitated by AI. The increased scale and uncertainty of large decisions during the transition from conventional to carbon-neutral grids present challenges that could be addressed by AI algorithms making decisions based on vast amounts of data as opposed to complex models, and paving the way for a carbon-neutral transition [53].

Furthermore, machine learning under AI has drawn significant interest, particularly in neural networks used to address complex and uncertain problems in energy systems. Diao et al. [54] created a convolutional neural network-based deep learning architecture and used it for the first time in a case study of supercritical CO2 Brayton cycle performance prediction. Zhu et al. [55] created a “decomposition-reconstruction-integration” thinking technique based on carbon constraints, eigenvalue transformations, and deep learning neural networks for short- and medium-term energy price forecasting, which has increased the accuracy of energy price forecasting. The carbon neutrality contribution of the existing forests planned afforestation, and forest nurturing operations in China from 2021 to 2060 was predicted using a combination of backpropagation neural networks, biomass conversion factor approaches, and logistic models [56]. Chen et al. [57] investigated a BP neural network learning algorithm-based carbon emission management and prediction method for electric cars to achieve pre-mass production prediction and in-use vehicle carbon emission analysis, which serves as a crucial reference for the precise management of vehicle carbon emissions. Yang and Liu [58] suggested a hybrid projection to examine China’s potential to become carbon neutral in the years 2020–2060, this projection combines the Elman Neural Network (ENN) and Sparrow Search Algorithm (SSA). With the development and application of AI, future studies in the domain of carbon neutrality should focus on the intricate integration of AI with carbon neutrality, which is a significant and popular research area.

#### 4.3.3 Carbon neutrality in the post-new crown epidemic era

Measures such as the closure of international borders, restrictions on the movement of people in their homes, and restrictions on travel or assembly during the COVID-19 crisis have had a serious economic and social impact [59]. The changes brought about by the epidemic have had a negative effect on industry and supply networks, the dynamics of energy conversion, and advancement in the application of technology [60]. According to the research that is currently available, “sequestration” measures implemented by a number of nations during the early years of the COVID-19 epidemic altered global energy demand patterns and had a significant impact on CO2 emissions, with significant reductions in carbon emissions over the same period [61]. Yet, the temporary carbon reductions brought on by the epidemic phase may result in a rise in carbon emissions in the wake of a quick economic rebound. Gribkova and Milshina [62] identified the primary strategies for the post-pandemic energy transition in numerous nations by forward-looking methods like trend tracking, case studies, and the STEPE methodology for barrier analysis. Yang et al. [63] believed that changes to the energy consumption mix will be the primary means of achieving carbon intensity targets in the post-epidemic future. Shah et al. [64] suggested that a switch to sustainable energy is more capable than ever of mitigating future climatic catastrophes by limiting carbon emission for Pakistan in the context of a green economic recovery following COVID-19. These studies serve as a crucial point of reference for the global objective of carbon neutrality in the post-epidemic era, and future study on how to meet objectives of decreasing climate change and ensuring energy security and efficiency without severely impeding economic growth is crucial [65].

#### 4.3.4 Metaverse and carbon neutrality

The metaverse is a new type of human society that blends the actual and fictional worlds with a high level of digitalization and intelligence [66]. Its advent has created new prospects for carbon neutrality. The digital economy, which is closely connected to the metaverse, will drastically cut down on the amount of energy used in real economic activity and, to some extent, alter people’s traditional ways of living and producing. Digitalization and intelligence will permeate the industrial system and chain involved in reducing carbon emissions in the metaverse society, enabling carbon neutrality in all respects, from energy substitution at the front end to energy conservation and emission reduction at the middle end to recycling at the back end. The power production system is one of the most common application scenarios for the integration of metaverse and carbon neutrality, and with the new power system suggested, the development of a metaverse power system is a trend for the future. By some technologies such as digital twins and IoT, it is possible to identify and analyze the energy storage system in the metaverse and elevate its management and control from simulation-based to metaverse-driven intelligent control [67]. Carbon neutrality is a green revolution in the supply chain and efficiency improvements in the consumer sector [68], and the metaverse has taken on the responsibility of lowering energy usage in the new economy. Therefore, future studies should broaden the use of the metaverse to additional energy-intensive businesses and scenarios, offering a robust theoretical and practical basis for the application and reform of the metaverse in a variety of industries.

## 5 Conclusion

The goal of reviewing journal papers on carbon neutrality is to identify some aspects of the issue that have not yet been investigated or may be examined more deeply and to address the three questions posed. With the aid of the software Vosviewer and Bibliometrix, this paper presents a comprehensive and systematic analysis of the literature on carbon neutrality. It also lays the groundwork for future research by providing a quantitative and visual review and discussion of the results that have been published on carbon neutrality.

First, we conduct an analysis and study of the 909 publications which are obtained from the WoS core database, looking at the authors, publications, countries/regions, journals, keywords, and research hotspots. The first thing to note is that since 2020, there has been a marked rise in the number of publications on carbon neutrality. The top contributing authors and institutions in the world are dispersed throughout, with China publishing the most articles, followed by the US, and developing nations conducting less research. Seven of the top ten papers in terms of citations are in the discipline of chemistry, while two are in the field of environmental science, including research hotspots such carbon neutral fuel research and carbon capture technology development. Second, we examine the relationships within the collaborative network and discover that the top three authors in terms of publications, Umar, Muhammad and Adebayo, Tomiwa Sunday, had established a substantial collaboration network, and China and the US are at the center of a global network of cooperation in terms of national cooperation. What’s more, “Sustainability”, “Journal of Cleaner Production”, and “Journal of Environmental Management” are the three journals with the most publications out of the 353 that contributed to the 909 papers that were chosen. “Journal of Environmental Management” has received the most citations and has a large impact in the field. The top 5 publishing research institutions are all located in China, and there is a strong network of collaboration between institutions, with Tsinghua University and the Chinese Academy of Sciences serving as the key nodes. Additionally, “carbon emissions,” “renewable energy technologies,” “carbon capture and storage,” “carbon conversion,” and “carbon conversion” are identified as hot and trending research topics through keyword co-occurrence analysis, word cloud analysis, and cluster analysis. The knowledge gleaned from the bibliometrics of the literature is then summarized, discussed, and new directions for research are suggested. The research hotspots and main routes to carbon neutrality are carbon replacement, carbon reduction, and CCUS, according to the bibliometric analysis. This paper discusses the above four hotspot and offers four directions for further investigation.

There are still many shortcomings in this paper. The WoS core collection only contains English-language publications, so keyword searches might not turn up all relevant and significant papers. Additionally, the search strategy and selection criteriaare subjective, which could have an impact on the analysis’s outcomes.

## Funding

This work was supported in part by the National Social Science Fund of China under Grant (22FGLB005), the Humanities and Social Science project of the Ministry of Education of China under Grant (21YJC630030), the Sichuan Science and Technology Program under Grant (2022JDR0305), and the Funds for Sichuan University to Building a World-class University under Grant (2021CXC21).

## Author contribution statement

Xunjie Gou; Xinru Xu; Weike Zhang; Yunying Zhao; Zeshui Xu: Conceived and designed the experiments; Performed the experiments; Analyzed and interpreted the data; Contributed reagents, materials, analysis tools or data; Wrote the paper.

## Data availability statement

Data will be made available on request.

## Declaration of competing interest

The authors declare that they have no known competing financial interests or personal relationships that could have appeared to influence the work reported in this paper.
